# Promoting rapid and sustained adoption of biofortified crops: What we learned from iron-biofortified bean delivery approaches in Rwanda^☆^^☆☆^

**DOI:** 10.1016/j.foodpol.2018.11.003

**Published:** 2019-02

**Authors:** Kate Vaiknoras, Catherine Larochelle, Ekin Birol, Dorene Asare-Marfo, Caitlin Herrington

**Affiliations:** a Virginia Tech, Department of Agricultural and Applied Economics, 250 Drillfield Drive, 306A Hutcheson Hall, Blacksburg, VA 24061, USA; b Virginia Tech, Department of Agricultural and Applied Economics, 250 Drillfield Drive, 315 Hutcheson Hall, Blacksburg, VA 24061, USA; c HarvestPlus/International Food Policy Research Institute, c/o IFPRI, 1201 Eye St., NW, Washington, DC 20005-3915, USA

**Keywords:** Biofortification, Iron-biofortified beans, Duration analysis, Adoption, Rwanda

## Abstract

Micronutrient deficiencies, also known as hidden hunger, affect two billion people worldwide, curtailing their ability to lead healthy, productive lives. Biofortified staple crops, bred to be rich in micronutrient content, are a cost-effective and scalable solution to alleviating micronutrient deficiency, particularly among rural households who consume what they produce. Delivery of biofortified planting material in Rwanda began in 2012, and it is important to learn from the efforts undertaken to date to inform the design of higher impact – lower cost delivery strategies for scaling up these crops. In this paper, we use a nationally representative household survey of bean producers and delivery data from seven consecutive seasons and apply duration analysis to estimate the impact of different delivery approaches on household time to adoption, disadoption and readoption of iron-biofortified beans in Rwanda. Proximity to formal delivery via sales of small packets of planting material quickens adoption and readoption, while delivery of larger quantities of planting material to small-scale producers within a village slows disadoption of iron-biofortified beans. Informal dissemination within social networks and access to extension are also major drivers of rapid adoption. In addition, households whose main decision maker for bean production is a woman, has some formal education, and more years of experience growing beans disadopt iron-biofortified beans more slowly than other households. These findings provide evidence that current efforts to promote iron-biofortified crops have been successful and are expected to inform future development of sustainable and cost-effective delivery models for biofortified crops in Rwanda and elsewhere.

## Highlights

• About 29% of households in Rwanda have grown an iron-biofortified bean variety.
• Formal delivery approaches of iron-biofortified beans increase adoption speed.
• Diffusion via social networks is a major driver of rapid adoption of iron-biofortified beans.
• Women farmers disadopt iron-biofortified bean varieties more slowly than do male farmers.

## 1 Introduction

Over a quarter of the world’s population suffers from micronutrient malnutrition, also known as hidden hunger, which can result in poor health, stunted growth, and decreased mental capacity, leading to productivity losses and lower lifetime earnings (Alderman et al., 2006, FAO, 2013). The cost of undernutrition and micronutrient deficiency is estimated at up to 3 percent of global GDP, which corresponds to an economic loss of up to $2.1 trillion per year (FAO, 2013, FAO, 2014). In the Copenhagen Consensus 2008, an expert panel ranked three micronutrient interventions in the top-five best investments to foster economic development in low-income countries (Copenhagen Consensus Center, 2008). These included providing vitamin and mineral supplements mainly targeted to children and pregnant women, fortification of food with micronutrients during processing, and biofortification, a process by which staple food crops are bred to have higher micronutrient content.

Randomized control trials have proven the efficacy of iron-biofortified crops in improving iron deficiency and functional outcomes. Studies conducted in Mexico and Rwanda found that consumption of iron-biofortified beans for just a few months improved iron status (Haas, 2014, Haas et al., 2016). Finkelstein et al. (2017) conducted a meta-analysis using efficacy trial data from three iron-biofortified crops: bean, rice, and millet, and found iron-biofortification to be effective in improving iron status, particularly for those who are iron-deficient. Moreover, iron-biofortified beans were found to have a significant effect on cognition: iron-deficient women who ate biofortified beans experienced improved memory and ability to pay attention (Murray-Kolb et al., 2017), key skills for optimal performance at school and work. The study also measured physical performance and results suggest improvements in iron status were accompanied by a reduction in time spent in sedentary activity (Luna et al., 2015).

Rwanda Agriculture Board, in collaboration with the International Center for Tropical Agriculture and HarvestPlus, developed and released four iron-biofortified bean varieties in Rwanda in 2010 and six additional varieties in 2012. Rwanda was identified as top-priority for investment in iron-biofortified bean breeding and delivery due to the importance of bean production and consumption in the country, and the significant rate of iron deficiency which can be alleviated through iron-biofortification of beans (Asare-Marfo et al., 2013). Over 90% of rural households grow bean (Asare-Marfo et al., 2016a). The crop is grown in both agricultural seasons (Seasons A and B^1^) and across Rwanda’s ten agro-ecological zones, which vary by soil type, altitude, terrain, and rainfall. Bean is a staple food in all zones (USAID and FEWS NET, 2011) and contributes 32% of calorie and 65% of protein intake (CIAT, 2004, Mulambu et al., 2017).

Intensive dissemination of iron-biofortified bean varieties began in 2012. Several delivery approaches were used including sales through authorized agrodealers, direct marketing by the HarvestPlus Rwanda country team in local markets, and exchange of local variety grains for iron-biofortified bean seeds. Informal dissemination also occurred through social networks. As a result, approximately half a million households grew an iron-biofortified bean variety for at least one growing season between 2010 and 2015 (Asare-Marfo et al., 2016a).

The objective of this study is to determine the effects of formal delivery and informal dissemination on the speed of adoption, disadoption, and readoption of iron-biofortified bean in Rwanda. This research contributes to the literature on adoption of improved crop varieties in three ways. First, it is one of the few studies on adoption of biofortified crops. Improved varieties are bred to increase productivity while biofortified crops, in addition to their yield gains, offer nutritional benefits. Thus, reasons for adopting biofortified crops may differ from those for other improved varieties. As more biofortified crops are released, it is important to identify factors that drive adoption. We also examine the determinants of disadoption and readoption to identify factors that lead to sustained production, since for biofortification to be successful in alleviating hidden hunger, biofortified crops must be produced and consumed in sufficient quantity over long periods of time.

Second, we consider adoption as a dynamic and sequential decision-making process by which households gather new information over time and in each growing season decide whether to begin, continue, stop, or start again the cultivation of an iron-biofortified bean variety. We employ duration models to identify factors that influence the time it takes households to adopt, disadopt, or readopt iron-biofortified beans. These models account for the effects of time-varying variables, control for time dependence in decision making, and avoid bias that occurs from measuring adoption at only one point in time (Keifer, 1988). It is important to understand factors that shorten the time until households adopt a biofortified crop and lengthen the number of seasons they grow it. Nutrient-deficient households require greater intake of micronutrients quickly and consistently, especially those with young children as poor nutrition at an early age can have irreversible consequences leading to fewer earning opportunities throughout life, perpetuating the vicious cycle of poverty (Alderman et al., 2006). Moreover, rapid adoption also means a higher rate of return on investment in biofortification, improving the cost-effectiveness of the technology and putting policy makers in a better position to justify the investment.

Finally, this study provides evidence on the impact of different delivery approaches for biofortified crops and the role of informal dissemination in improving the speed of adoption. Findings will be incorporated into future delivery of biofortified crops for faster, more cost-effective and sustainable scaling-up of these crops.

The next section of this paper provides background information on iron-biofortified bean delivery in Rwanda. Section three explains our conceptual framework and empirical model of farmer decision making over time, and describes the data, explanatory variables, and estimation strategies. Section four provides descriptive and analytical results. The final section concludes with implications for policy and program design for biofortification.

## 2 Iron-biofortified bean varieties and delivery approaches in Rwanda

In addition to their high iron content, the ten iron-biofortified varieties are also high-yielding^2^ and resistant to pests and diseases. The varieties have different agronomic and consumption characteristics to accommodate diverse agro-ecological conditions and consumer preferences, and were developed to cater to the traits that female farmers value (Mulambu et al., 2017). Of the ten iron-biofortified bean varieties released, eight are of climbing type and two are bush varieties. Climbing bean varieties are higher yielding than bush bean varieties, grow upright, and require the use of stakes to achieve their high yield potential. Characteristics of the ten varieties are presented in Table S.1 in the supplementary material.

Formal delivery of iron-biofortified bean varieties began in season 2012B and intensified over the following seasons. Contracted seed multipliers produce certified seed from iron-biofortified bean foundation seed. Farmers can purchase the certified seeds through authorized agrodealers in packages ranging from 1 to 50 kg, and in local markets in small packets of 200–500 g; as per the sales records, this direct marketing approach reached a quarter of a million farmers by 2015, the largest number of any delivery approach (Mulambu et al., 2017). To reach more farmers, in 2013A, HarvestPlus and partners initiated a delivery mechanism called payback. Under this mechanism, farmers received iron-biofortified bean seed under the condition that they would give an agreed upon proportion of their harvested grain to the program. In 2015A, payback was replaced by the seed swap scheme, under which farmers traded their local bean grain (which was to be used as planting material) for iron-biofortified bean seed. By 2015, the payback/seed swap mechanism delivered the greatest quantity of seeds of any delivery approach. Like most certified seed in Rwanda, each delivery approach sells or provides seed to farmers at a subsidized price (Mulambu et al., 2017). RWR2245, a bush variety, has been the most widely disseminated, making up between 71% and 86% of total disseminated seed each season since 2013A, followed by MAC44, a climbing variety, which made up 10% to 29% of total disseminated seed each season (Asare-Marfo et al., 2016b).

**Fig. 1:**
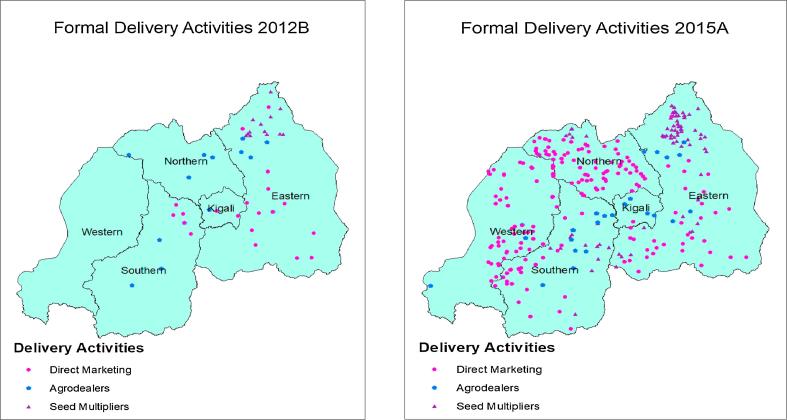
Formal delivery activities in 2012B and 2015A.

**Fig. 2:**
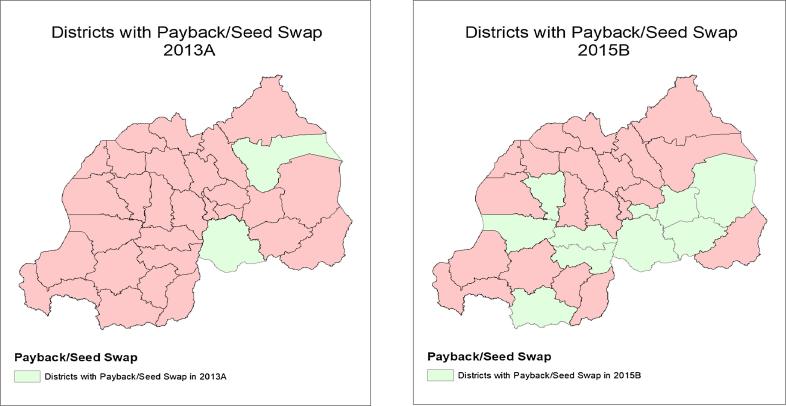
Districts with Payback/Seed Swap in 2013A and 2015B.

Fig. 1 shows the locations of seed multipliers, agrodealers, and direct marketing in season 2012B- the first season of intensive delivery-, and 2015A -the last season for which geolocations of direct marketing are available. In 2012B, seed multipliers were located in the northern part of the Eastern province, where land availability is greatest; by 2015A they were still concentrated in this area, but had also expanded to the remainder of the Eastern province as well as to the Southern and Northern provinces. The area reached by agrodealers also expanded during this period. In 2012B, agrodealers were in all provinces except the Western province, but were sparsely distributed. By 2015A, they were in all provinces, and with greater concentration in the Eastern, Southern, and Kigali provinces. Finally, direct marketing started in the Eastern and Southern provinces in 2012B and by 2015A had spread to all provinces. The number of districts in which payback and seed swap mechanisms operated increased between 2013A and 2015B (Fig. 2). In 2013A, the first season payback was established, it operated in only two districts. By 2015B, seed swap was operating in ten districts.

## 3 Conceptual and empirical framework of adoption timing and data

### 3.1 Conceptual framework

We model adoption of agricultural technology as a sequential process that happens over growing seasons, similar to that of Ma and Shi (2015): households collect information about a technology, make an initial decision to use the technology, and then update their knowledge according to their own experiences. In each subsequent growing season after adoption, households decide whether to continue to use or disadopt the technology; if they disadopt, they then decide in each following season whether to start using the technology again.

The decision of household *i* to grow an iron-biofortified bean variety *j*, which is part of the set of all available bean varieties *J*, at the start of each growing season *t* depends on the expected utility of growing the variety in that season $U_{\mathrm{ij}}\left(t\right)$ compared to the expected utility of growing all alternative varieties $U_{\mathrm{iJ}}\left(t\right)$, and constraints faced by the household related to income and awareness of the variety. If $\left(U_{\mathrm{ij}}\left(t\right)-U_{\mathrm{iJ}}\left(t\right)\right)=v_{\mathrm{ij}}\left(t\right)>0$ and constraints are not binding, then household *i* will grow variety *j* in season *t*.

The value of $v_{\mathrm{ij}}\left(t\right)$ depends on season *t* expected costs and benefits of growing variety *j*. The household accrues monetary and opportunity costs of gathering information about biofortified varieties and obtaining the planting material. Expected benefits include the yield gain and other production advantages of the new variety compared to other varieties, as well as their superior nutritional qualities. The value of $v_{\mathrm{ij}}\left(t\right)$ and constraints to adoption vary across household and village characteristics $\left(X_{\mathrm{it}}\right)$ and shift over time as formal iron-biofortified bean delivery approaches $\left(F_{\mathrm{it}}\right)$ expand and change locations and informal dissemination through social networks $\left(I_{\mathrm{it}}\right)$ increases.

Formal delivery approaches $\left(F_{\mathrm{it}}\right)$ and informal dissemination of iron-biofortified bean varieties $\left(I_{\mathrm{it}}\right)$ through social networks will influence adoption decisions in two ways; first, by increasing the likelihood that a household is aware of the variety and second, by reducing the costs of adoption by making planting material more easily accessible. Additional household and village characteristics $\left(X_{\mathrm{it}}\right)$ that form a household’s resources, knowledge and preferences, will influence adoption through their effects on income constraints, probability of awareness, and costs and benefits of adoption.

### 3.2 Duration analysis of adoption, disadoption, and readoption

We use discrete duration analysis to empirically model the sequential adoption process. Duration analysis incorporates the time-dependence of decision making, and can also account for the effects of time-varying covariates. The outcome of interest of duration models is the length of a spell, *T_ikj_*, where *k* denotes spell order. Thus, we break the sequential adoption process of each iron-biofortified variety into three spells. The first spell (*T_i1j_*) starts the season iron-biofortified bean varieties were first disseminated (i.e. 2012B) and ends the first season household *i* adopts variety *j*. The second spell (*T_i2j_*) starts the season after household *i* adopted variety *j* and ends the season it disadopts that variety. The third spell (*T_i3j_*) begins the season after household *i* disadopts variety *j* and ends the season it readopts that variety. Additional spells exist for households that cycle in and out of growing variety *j*.

We are interested in the lengths of the spells *T_i1j,_ T_i2j_*, and *T _i3j_*. The cumulative distribution function of *T_ikj_* represents the probability that spell *T_ikj_* ends prior to season *t_ikj_*:

(1) $$F\left(t_{\mathrm{ikj}}\right)=\int_{0}^{t_{\mathrm{ikj}}}f\left(t_{\mathrm{ikj}}\right)dt=Pr\;\left(T_{\mathrm{ikj}}\leq t_{\mathrm{ikj}}\right)$$

The distribution of *T_ikj_* can also be represented by the survival function, which is the probability that *T_ikj_* ends after *t_ikj_*:

(2) $$S\left(t_{\mathrm{ikj}}\right)=1-F\left(t_{\mathrm{ikj}}\right)=Pr\;\left(T_{\mathrm{ikj}}>t_{\mathrm{ikj}}\right)$$

Duration analysis allows the estimation of the hazard rate, $h\left(t_{\mathrm{ikj}}\right)=\frac{f\left(t_{\mathrm{ikj}}\right)}{S(t_{\mathrm{ikj}})}$, which is the probability that the spell ends on a season *t_ikj_*, given that it has not already ended. We model the hazard rate empirically using a proportional hazard model, which allows us to evaluate the effects of covariates on the speed of adoption ($h_{i1j}$), the speed of disadoption, given adoption, ($h_{i2j}$), and the speed of readoption, given disadoption, ($h_{i3j})$. The hazard rate for household *i* and bean variety *j* is:

(3) $$h_{\mathrm{ikj}}\left(t_{\mathrm{kj}},{F_{\mathrm{it}},I_{\mathrm{it}},X}_{\mathrm{kit}},\beta_{\mathrm{kj}}\right)=h_{0kj}\left(t_{\mathrm{kj}}\right)*\exp\left(F_{\mathrm{it}}+I_{\mathrm{it}}+X_{\mathrm{kit}}\right)\beta_{\mathrm{kj}}$$

where $h_{0kj}$ is the baseline hazard function, which models the time dependence of adoption, disadoption and readoption decisions, *t_kj_* represents the number of growing seasons that have passed since the spell began, $F_{\mathrm{it}}$ is a vector of formal delivery variables, $I_{\mathrm{it}}$ is a vector of informal dissemination variables, and$X_{\mathrm{it}}$ is a vector of household and village characteristics. Finally, $\beta_{\mathrm{kj}}$ is the vector of parameters to be estimated that captures the effects of covariates on the hazard rate.

Due to low adoption rates of some of the iron-biofortified bean varieties, we pool the varieties together to estimate the following proportional hazard models:

(4) $$h_{\mathrm{ikj}}\left(t_{k},{F_{\mathrm{it}},I_{\mathrm{it}},X}_{\mathrm{kit}},V_{j},\beta_{k}\right)=h_{0k}\left(t_{k}\right)*\exp\left(F_{\mathrm{it}}+I_{\mathrm{it}}+X_{\mathrm{kit}}+V_{j}\right)\beta_{k}$$

where $V_{j}$ is an indicator variable for individual iron-biofortified bean variety. This variety fixed effect allows us to capture differences in the hazard rate associated with each variety, proxying for varietal traits and differences in availability of varietal planting material.

### 3.3 Data

To estimate the proportional hazard model in Eq. (4), this study uses nationally representative data of rural bean producers in Rwanda collected in two stages. In the first stage, 120 villages were randomly selected and all households in the selected villages were interviewed as part of a brief listing exercise. The goal of the listing exercise was to collect information about iron-biofortified bean adoption and inform the second stage of the data collection process. To facilitate bean varietal identification, households were shown a seed sample of one iron-biofortified bean variety and asked whether they had heard of the variety, grown it, the season they first adopted, and whether they had grown the variety in each subsequent season. The enumerators repeated this process for the nine remaining varieties. The listing exercise was conducted in May and June 2015 (i.e. season 2015B) and included 19,575 households (Asare-Marfo et al., 2016a).

In the second stage, 12 households per village were re-interviewed in greater depth in September-October 2015, after harvesting of the same season. When possible, six iron-biofortified bean adopters who grew an iron-biofortified bean in 2015B and six non-adopters were selected randomly in each village. In villages with fewer than six iron-biofortified bean adopters, all adopters were selected and non-adopters were randomly selected to obtain a total of 12 households. Enumerators interviewed the household member responsible for bean production decision making during season 2015B about household demographics and composition, bean farming decision making, asset ownership, bean production and consumption, and iron-biofortified bean adoption history from 2012B to 2015B.

A community survey, conducted along with the main household survey, was administered to key informants including the village leader to gather information on village characteristics, services and amenities related to market access, extension, and the presence of formal iron-biofortified bean delivery approaches in the village. One village surveyed during the listing exercise had no bean growers and thus was not considered for the household survey and one household had missing data. Therefore, the final sample includes 1396 households, located across 119 villages, and 29 districts.

We use household geographical coordinates, community survey data, and locations of seed multipliers and delivery approaches to estimate farmer proximity and access to iron-biofortified bean seeds for growing seasons 2012B-2015B. We compute the distance between households and agrodealers, and households and seed multipliers for each growing season. To capture proximity to promotion and sales locations of iron-biofortified beans, we count the number of direct marketing approaches in a given sector (an administrative unit) in each season. We cross-reference community survey responses with delivery records to determine whether payback and seed swap operated in each sampled village between 2012B and 2015B.

### 3.4 Variables

The dependent variable is a binary variable that is equal to one if spell *k* ended and zero otherwise for household *i* variety *j* in season *t.* This allows us to capture the total number of seasons between the beginning and end of a spell. We expect that the probability of adoption will increase as time passes, as households have more time to gain knowledge and awareness of the varieties. After several seasons of growing, households may wish to replace their bean planting material and try new varieties, so we expect that the probability of disadoption will also increase over time. We expect the probability of readoption to decrease over time. Some disadoption that is followed by subsequent readoption may be due to seasonality of bean growing, in which case readoption would occur after just one season of discontinued use. If readoption does not occur after one season, it could indicate that the realized benefits of the variety to the household were lower than expected, making readoption less likely.

The vector of formal delivery approaches, $F_{\mathrm{it}}$, includes the following time-varying variables: number of direct marketing approaches in the household’s sector, whether payback/seed swap occurred in one’s village, distance from the household to the nearest agro-dealer of iron-biofortified bean seeds, and distance to the nearest seed multiplier (Table 1). Proximity to formal delivery approaches improves access to and information about iron-biofortified bean varieties, which should reduce the length of time until adoption and readoption, and increase the amount of time until disadoption. While seed multipliers do not disseminate iron-biofortified bean seed to farmers directly, households living near the supply of seeds may face lower opportunity costs of obtaining information about and gaining access to iron-biofortified bean varieties.

**Table 1:** Variable names and descriptions for covariates of adoption, disadoption, and readoption models.

Variable name	Variable description	Time-varying
*Formal delivery*		
Direct markets	Number of direct marketing approaches in the sector	Yes
Payback	1 = someone in village has participated in payback	Yes
Seed swap	1 = someone in village has participated in seed swap	Yes
Agrodealers	Distance to nearest agrodealer selling iron-biofortified bean seeds, in km	Yes
Multipliers	Distance to nearest seed multiplier of iron-biofortified bean seeds, in km	Yes

*Informal dissemination*		
Adoption rate	Previous-season village adoption rate of iron-biofortified beans	Yes

*Household and village characteristics*		
Sex	1 = respondent is a female	No
Education	Education level of respondent: 0 = no schooling; 1 = some primary education; 2 = some secondary education or more	No
Experience	Bean farming experience of the respondent, in years	Yes
Household size	Number of household members	Yes^a^
Share 0–5	Proportion of household members age 0–5 years	Yes^a^
Share women	Proportion of household members that are women of child-bearing age (15–49 years)	Yes^a^
Wealth tercile	Wealth index created using polychoric principal components analysis expressed in tercile (measured using 2015B assets)	No^b^
ag. equipment	Count of agricultural equipment^c^ owned in 2015B	No^b^
Cultivated land	Land cultivated in 2015B for all crops, in 100 m^2^	No^b^
City distance	Distance to nearest city of at least 50,000 people, in km	No
Extension access	% of households in the village who obtain information from agricultural extension agents	No
Social seed source	0 = first planting material came from local markets, RAB, or HarvestPlus (formal channels); 1 = first planting material came from neighbors, relatives or friends (social channels)	No
Zone	Agro-ecological zone (1–10)	

*Variety*		
Variety	Categorical variable to distinguish between the iron-biofortified bean varieties (RWR2245^d^, MAC44, RWV3316, RWV3317, RWV1129, RWR2154^d^, CAB2, RWV2887, MAC42, RWV3006); base = RWR2245	No

a Values for previous seasons were calculated by subtracting backward from household members’ ages in 2015. This requires the assumption that no one died, left the household, or entered the household between 2012 and 2015.

b Although these variables are likely to change over time, we only collected data on their 2015 values; therefore, in our estimations, these variables are not time-varying.

c Includes plough, wheelbarrow, machete, shovel, pick, and sprayer.

d Variety is a bush variety (all other varieties are climbing).

We define informal dissemination, $I_{\mathrm{it}}$, as the process by which households gain access to the new technology through their social networks. We use the village level adoption rate in the previous season, calculated from the listing exercise data, as a proxy for the extent of information about iron-biofortified bean and availability of the technology in one’s social network. Learning via social information networks has been found to significantly influence adoption behavior (Beyene and Kassie, 2015, Matuschke and Qaim, 2009, Wollni and Andersson, 2014). Social networks improve access to information, but can also lead to free-riding and strategic delay as households wait for others to gather information about the technology (Bandiera and Rasul, 2006, Beyene and Kassie, 2015, Michelson, 2017).

Few studies have examined the role of social networks on disadoption, and findings are mixed (Lambrecht et al., 2014, McNiven and Gilligan, 2012, Michelson, 2017, Moser and Barrett, 2006). Once a farmer has grown an iron-biofortified bean variety, learning from other farmers in the village may become less valuable, though he/she may have better access to planting material when there are several adopters within his/her social network. We therefore expect that the previous season’s village-level adoption rate will either have no effect or will lengthen the time to disadoption, and will have either no effect or will shorten the time until readoption.

The vector of household and village characteristics, $X_{\mathrm{it}}$, includes access to extension, household wealth and composition, education, bean farming experience and gender of the respondent, market access, whether the planting material came from a social (informal) or formal source, and agro-ecological zone. Access to agricultural extension (measured at the village level to avoid endogeneity), as an approximation by the village leader of the percentage of households who currently use extension services, is expected to shorten the time until adoption and readoption, and lengthen the time to disadoption, by increasing household ability to access and to some extent process information (Beyene and Kassie, 2015, Feder et al., 1985, Foster and Rosenzweig, 2010, Wollni and Andersson, 2014). In addition, extension agents in Rwanda may teach households the benefit of planting single-variety bean seeds, rather than recycled bean grain or purchased mixed-variety grain, which is commonly practiced (HarvestPlus, 2017). These households may also be more likely to obtain yield gains in line with expectations, making them more likely to continue growing the variety (Lambrecht et al., 2014).

Household wealth, measured using a wealth index, a count of agricultural equipment owned and land area cultivated, is expected to increase the speed of adoption since wealth is associated with greater access to resources and a better ability to bear the risk associated with adopting a new technology (Feder et al., 1985, Foster and Rosenzweig, 2010, Nazli and Smale, 2016). It may also increase the time to disadoption and reduce the time to readoption by reducing income constraints that could prevent households from purchasing new planting material. Household composition includes household size, the proportion of household members made up of women of childbearing age, and the proportion of household members made up of children under five years of age, two of the most vulnerable groups to micronutrient deficiencies. Households with larger shares of women and children may face greater constraints to adopt due to lower labor availability but could also be more likely to adopt quickly and continuously since women and children are the most likely to benefit from the consumption of iron-biofortified beans. This could also make households adopt more continuously (i.e. disadopt later) and be more likely to readopt.

Education and years of bean farming experience of the respondent are expected to speed adoption and readoption while slowing disadoption by improving household access to information and ability to process that information, similar to the expected role of extension. Educated respondents may be more aware of the nutritional needs of their families and the nutritional benefits of biofortified crops, which would make them more likely to adopt quickly and continuously. Both education and farming experience may also increase the household’s ability to achieve the yield potential of iron-biofortified beans. Gender has been found to affect production preferences and access to resources (Doss, 2001), although it is difficult to predict the relationship between gender and adoption of iron-biofortified beans. Women may be more resource-constrained but may also value the traits of iron-biofortified beans, particularly since they were developed to incorporate women’s preferences (Mulambu et al., 2017).

Market access is measured by distance to the nearest city of 50,000 inhabitants. As of 2012, there were five such cities in Rwanda, with at least one in each province except for the East (Bundervoet et al., 2017). Proximity to cities of this size can facilitate access to information and reduce the cost of input acquisition, promoting rapid adoption.

The models examining disadoption and readoption also include a binary variable indicating the source of planting material in the first season the variety was grown. This variable is equal to one when seeds came from a social, informal source (i.e. a friend, relative or neighbor) and zero if seeds were obtained from a formal source (i.e. local markets, RAB or extension, or a HarvestPlus delivery approach). Certified seed from formal delivery tends to be a higher quality planting material, providing higher yield than second-generation planting material (i.e., grain used for planting). This could make households who first received planting material from social, informal sources more likely to disadopt quickly. Alternatively, when households obtain planting material from neighbors, friends, or relatives, the variety is likely well-suited to their growing conditions, and the households can benefit from this person’s experience with the variety. In this case, households whose first source of iron-biofortified beans was a social, informal one may be less likely to disadopt quickly. In addition, households that disadopted may be better able to re-access planting material if their initial planting material was from a social source, making them more likely to readopt quickly.

Variety fixed effects are included as proxy variables for differences in varietal characteristics and availability. They allow us to evaluate whether the hazard rates of adoption, disadoption, and readoption vary by variety after holding other factors constant.

Finally, agro-ecological zone fixed effects are included in all models to control for differences in agricultural potential.

### 3.5 Estimation and data limitations

Discrete duration models are estimated through maximum likelihood techniques. We use the complementary log-log model because its exponentiated model coefficients can be interpreted as hazard rates (Jenkins, 2008). We determine whether unobserved heterogeneity (e.g. unobserved managerial skills of the farmer), or frailty, influences the time to adoption, disadoption and readoption by including household random effects. If frailty is present and not accounted for, estimated coefficients *β_k_* can be biased (Keifer, 1988). We test the null hypothesis of no unobserved heterogeneity using a likelihood ratio test.

The time dependence variable *t_k_* enters each proportional hazard model in Eq. (4) through the baseline hazard model, $h_{0k}$, which can take different functional forms. For each model, we estimate the three most common functional forms for discrete duration analysis: log time (log(*t_k_*)), cubic polynomial function of time (*t_k,_ t_k_^2^_,_ t_k_^3^*), and a piecewise-constant function of time, in which the variable *t_k_* enters Eq. (4) as a series of dummy variables pertaining to individual growing seasons^3^ (Jenkins, 2008). We identify the most appropriate functional form using the Akaike information criterion (AIC).

Standard errors are robust to heteroskedasticity and clustered at the village level. Because the sampling procedure oversampled adopters, we use sampling weights in all descriptive statistics and duration models, meaning that the findings are representative of bean producers in Rwanda.

Households enter spell *T_2j_* the season after spell *T_1j_* ends and they enter spell *T_3j_* the season after *T_2j_* ends. Because season 2015B is the last season for which we observe household adoption behavior, we are not able to estimate *h_2j_* for households who adopted that season. Likewise, we are unable to estimate *h_3j_* for households who disadopted in 2015B.^4^ Furthermore, households that adopted prior to 2012B, which make up 3.5% of our sample, cannot be included in the duration analyses because they completed their transition to stage two before the start of the intensive delivery mechanisms analyzed in this study.

Our data has two limitations in estimating coefficients *β_k_*. The first is that the adoption data is dependent on household recall and proper identification of the varieties. While varietal identification was supported with seed samples during the data collection process, households still may not accurately remember when they first began or stopped growing iron-biofortified bean varieties. However, the relatively short time frame between varietal dissemination and data collection, and the fact that most adopters adopted recently reduce the likelihood of recall bias. The second limitation is that several variables, such as asset ownership, which is part of the wealth index, and land area cultivated, are only measured in the season data were collected (2015B) while we are attempting to explain adoption behavior that occurred between 2012 and 2015. We must therefore assume that variables for which we do not have time-varying information (indicated in Table 1) did not change during this period. Fortunately, our main variables of interest, formal delivery approaches and informal dissemination, are time-varying.

## 4 Results

### 4.1 Descriptive statistics

**Fig. 3:**
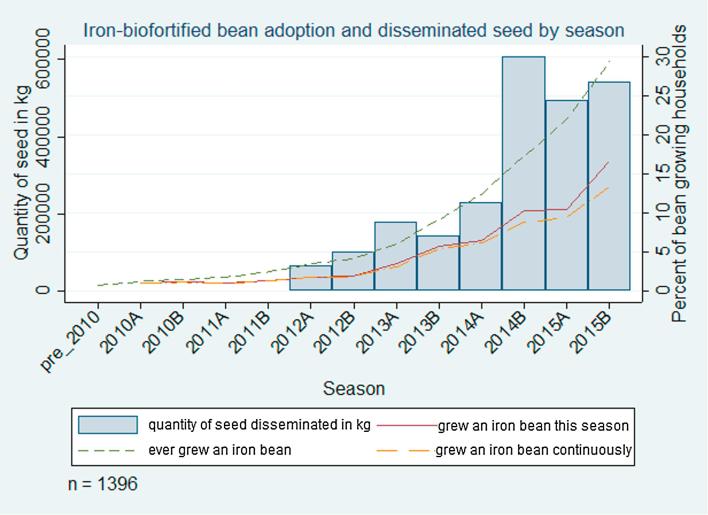
Iron-biofortified bean adoption and disseminated seed by season.

Prior to 2012B, less than 4% of the population grew an iron-biofortified bean (dotted green line in Fig. 3). Adoption increased steadily after 2012B, which corresponds to the beginning of intensive delivery efforts for iron-biofortified beans. By 2015B, about 29% of bean producers had cultivated at least one iron-biofortified bean variety in at least one season (dotted green line in Fig. 3). The increase in adoption over time is consistent with the pattern of seed delivery; 101,716  kg of seed were delivered in Rwanda in 2012B compared to 540,660 kg in 2015B, with a maximum of 606,696  kg delivered in 2014B (reflected in the large increase in adoption that season).

Households also disadopted iron-biofortified beans, as exhibited by the difference between the percentage of households who ever grew a variety (dotted green line in Fig. 3) and those who grew a variety that season (solid red line in Fig. 3). In 2015B, 17% of households grew an iron-biofortified bean variety, indicating that about 12% of households had disadopted. Some households readopted iron-biofortified bean varieties, explaining the difference between current growers (solid red line in Fig. 3) and continuous growers (dashed orange line in Fig. 3). About 13% of households growing an iron-biofortified bean in 2015B had done so every season since adopting, indicating that about 4% of households had readopted.

**Fig. 4:**
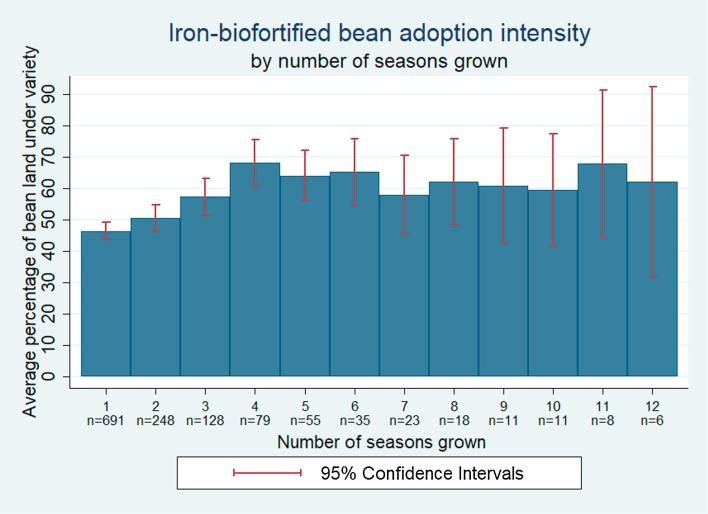
Iron-biofortified bean adoption intensity.

On average, adopters devote 51% of their land under bean cultivation to iron-biofortified varieties. This value varies with the number of seasons households have grown the variety, ranging from 47% of bean land area the first season to 68% the fourth season an iron-biofortified bean is grown (Fig. 4). This pattern holds when restricting the sample to only varieties grown four or more seasons; intensity of adoption of these varieties increases from 52% of bean area planted in the first season to 68% in the fourth season, after which it fluctuates from 58% to 68%. Households thus ramp up adoption intensity over the first four seasons they grow a variety and then stabilize intensity.

Most adopters have grown only one iron-biofortified bean variety; 14% have grown two and 1% have grown three. Of the households that grew more than one variety, 70% grew these varieties concurrently while 30% grew one, disadopted it, and grew another later.

**Fig. 5:**
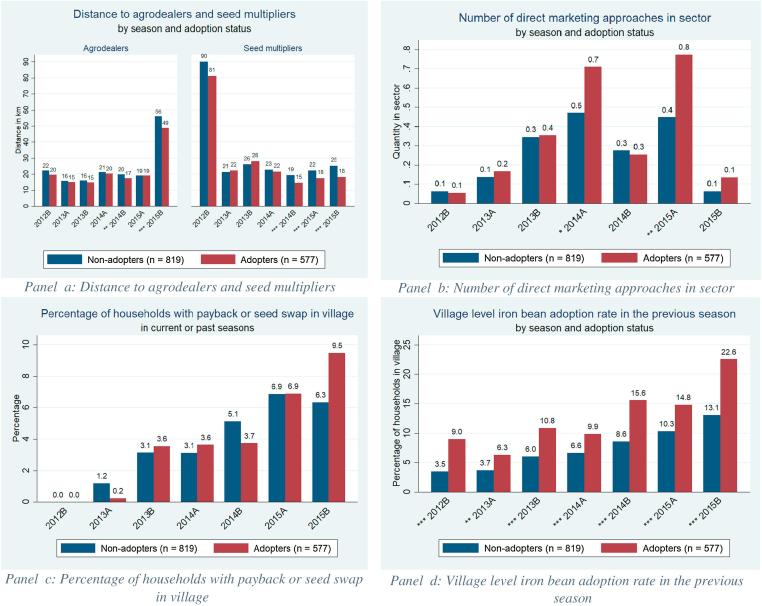
Descriptive statistics for formal and informal delivery approaches. Note: * = significance at 10%; ** = significance at 5%; *** = significance at 1%.

Summary statistics for delivery approaches, presented by season and adoption status, are given in Fig. 5. The average distance between households and agrodealers fluctuated between 15 and 22 km from 2012B to 2015A, and more than doubled in 2015B due to a lower number of authorized agrodealers in that season (panel a). On average, adopters live closer to agrodealers than non-adopters but the difference is significant only in 2014B and 2015B. The average distance between households and seed multipliers fell sharply after 2012B, and fluctuated between 15 and 28 km afterward (panel a). Adopters resided significantly closer than non-adopters to seed multipliers in 2014B, 2015A, and 2015B.

The average number of direct marketing approaches by sector increased steadily from 2012B to 2014A, fluctuated (peaking at 0.78 for adopters in 2015A and 0.47 for non-adopters in 2014A), and then fell in 2015B (panel b) due to a reduction in the efforts to promote and sell iron-biofortified seeds in local markets that season. The intensity of sales in local markets was significantly greater for adopters than non-adopters in 2014A and 2015A.

The proportion of households living in a village where payback/seed swap took place remained below 10% every season (panel c) and did not vary significantly by adoption status. The previous-season village level adoption rate increased in each season for non-adopters, but fluctuated for adopters from 6% in 2013A to 23% in 2015B (panel d). The previous-season adoption rate in the village was significantly higher for adopters than non-adopters in every season. Summary statistics of remaining covariates are presented in Table 2; adopters are defined as households who have ever grown an iron-biofortified bean variety.

**Table 2:** Descriptive statistics for covariates of adoption, disadoption, and readoption model.

Variable name	Adopters mean (SD) or %	Non-adopters mean (SD) or %	Statistical significance of differences in means
Gender (1 = female)	0.63 (0.48)	0.63 (0.48)	

*Education*			
No schooling	0.23 (0.42)	0.36 (0.48)	***
Some level of primary	0.66 (0.47)	0.58 (0.49)	
Some secondary or more	0.10 (0.30)	0.06 (0.23)	**
Bean experience (years)	25.71 (15.07)	27.90 (16.86)	*
Household size	5.08 (2.07)	4.74 (2.03)	
Share 0–5 years old	0.15 (0.16)	0.16 (0.18)	
Share women	0.25 (0.15)	0.24 (0.16)	

*Wealth tercile*			
1	0.30 (0.49)	0.40 (0.49)	***
2	0.31 (0.46)	0.33 (0.47)	
3	0.40 (0.49)	0.26 (0.44)	***
ag. equipment (nb)	1.34 (0.79)	1.19 (0.77)	**
Cultivated land (100 m^2^)	56.81 (83.44)	43.02 (72.95)	**
City distance (km)	37.96 (22.38)	36.59 (19.73)	
Extension access (%)	0.68 (0.26)	0.64 (0.28)	*
Social seed source (1 = yes)	0.41 (0.49)		

Number of observations	577^a^	819^a^	

* Significance at 10%.

** Significance at 5%.

*** Significance at 1%. Values for time-varying variables are given for 2015B.

a These values are the unweighted frequencies of adopters and non-adopters in the sample.

The respondent is, on average, significantly more educated among adopting than non-adopting households. Adopters are also significantly wealthier, own more agricultural equipment, and cultivate more land, on average, than non-adopters. Households that adopted more than one variety (not shown in the table) have more education, are wealthier, and cultivate more land than those who adopted only one variety. Other household and village characteristics do not vary significantly between adopters and non-adopters.

### 4.2 Model results and discussion

Results for the adoption, disadoption, and readoption models are presented in Table 3. Results are expressed as hazard ratios (exponentiated coefficients of the complementary log-log model). A hazard ratio greater (less) than one means that the variable makes the spell end faster (slower). Based on AIC, the piecewise-constant specification is the most appropriate to capture time dependence for the adoption and disadoption models, while the cubic polynomial time function slightly outperforms the piecewise-constant specification for the readoption model.^5^ For consistency, and because coefficients change very little over the different specifications, we present the piecewise-constant baseline hazard specification for all three models. The time dummy variables in all three models represent seasons that have passed since the respective spell began; for the adoption model, these refer to the number of seasons since the beginning of intensive delivery approaches in 2012B. For the disadoption (readoption) model, the time dummy variable is the number of seasons since adoption (disadoption) and is household-specific.

**Table 3:** Complementary log-log model results for adoption, disadoption, and readoption of iron-biofortified bean varieties.^d^

	Adopt		Disadopt		Readopt	
	Hazard Rate	(Robust Std. Err)	Hazard Rate	(Robust Std. Err)	Hazard Rate	(Robust Std. Err)
*Time dependence (base = 2012B/one season)*						
2013A/two seasons	2.655*	(1.353)	0.566*	(0.183)	0.034***	(0.037)
2013B/three seasons	4.197***	(2.077)	0.300***	(0.118)	0.140*	(0.158)
2014A/four seasons/four seasons or more	4.154***	(1.983)	0.103***	(0.060)	0.714	(0.524)
2014B/five seasons or more	6.505***	(3.414)	0.209**	(0.135)		
2015A	5.361***	(2.393)				
2015B	7.774***	(3.820)				
direct markets (# in sector)	1.208***	(0.047)	0.987	(0.034)	2.078**	(0.664)
payback (1 = in village)	0.889	(0.333)	0.385***	(0.105)	3.312	(3.484)
seed swap^a^ (1 = in village)	1.566	(0.535)				
agrodealers (km)	1.003	(0.004)	1.003	(0.004)	1.028***	(0.010)
multipliers (km)	0.998	(0.004)	0.991	(0.008)	0.994	(0.017)
village adoption rate	1.029***	(0.005)	1.007	(0.006)	1.028*	(0.015)
gender (1 = female)	1.103	(0.123)	0.646**	(0.116)	0.448*	(0.188)

*Education (base = no education)*						
some primary	1.450**	(0.226)	0.659**	(0.132)	1.017	(0.576)
some secondary or more	1.442*	(0.301)	0.333***	(0.115)	1.407	(1.551)
experience (years)	0.999	(0.004)	0.977***	(0.005)	1.020	(0.023)
household size	1.048	(0.033)	1.027	(0.041)	1.083	(0.155)
share 0–5	0.642	(0.219)	1.149	(0.510)	0.309	(0.528)
share women	1.150	(0.368)	0.801	(0.427)	3.524	(9.577)

*Wealth tercile (base = 1)*						
2	1.028	(0.134)	1.048	(0.190)	0.800	(0.572)
3	1.277*	(0.175)	0.890	(0.204)	2.080	(1.280)
ag. equipment	1.296***	(0.121)	1.081	(0.127)	1.557*	(0.412)
cultivated land (100 m^2^)	1.000	(0.001)	0.998	(0.002)	0.999	(0.003)
city distance (km)	0.998	(0.005)	1.003	(0.006)	1.029*	(0.016)
extension	1.008***	(0.002)	1.001	(0.003)	0.991	(0.010)
social source			1.113	(0.162)	0.868	(0.465)

*Variety (base = RWR2245*^b^*)*						
MAC44	0.319***	(0.072)	1.364	(0.266)	0.243**	(0.166)
RWV3316^c^	0.132***	(0.038)	0.739	(0.193)	0.161	(0.186)
RWV3317	0.072***	(0.019)	0.829	(0.266)		
RWV1129	0.061***	(0.030)	0.324**	(0.171)	1.400	(1.226)
RWR2154^b^	0.031***	(0.012)	0.828	(0.277)	0.614	(0.692)
CAB2	0.045***	(0.016)	0.687	(0.213)	1.559	(1.512)
RWV2887	0.032***	(0.011)	1.322	(0.568)	0.388	(0.448)
MAC42	0.045***	(0.017)	0.666	(0.362)	0.083***	(0.073)
RWV3006	0.057***	(0.018)	1.514	(0.490)	0.008***	(0.008)
N	96,197		683		333	

* Significance at 10%.

** Significance at 5%.

*** Significance at 1%.

a Seed swap had to be dropped from the disadoption and readoption models due to the low overlap between villages that had seed swap, villages that were sampled, and adopters in those villages.

b Bush variety.

c RWV3317 and RWV3006 had to be combined in the readoption model because RWV3317 perfectly predicted non-readoption.

d Previous versions of these models included variables regarding livestock ownership, household membership in a farmer’s association, the percentage of farmers in the village who sell beans in local markets, and a dummy variable indicating whether the household had heard the iron bean promotional song on the radio or seen the accompanying music video on television. These variables were removed due to lack of statistical significance. Removing them did not change the level of significance or magnitude of the coefficients for the delivery approach variables in the adoption or disadoption models, but increased the magnitude of the coefficient for direct marketing and reduced the size of the coefficient for previous season village adoption rate in the readoption model. When these variables were included, direct marketing was not statistically significant in the readoption model, while the adoption rate was significant at 1%. Most of these changes come from removing the variable indicating whether a household member belongs to a farmer’s organization.

We fail to reject the null hypothesis of no unobserved household heterogeneity for the adoption and disadoption models but not for the readoption model.^6^ Because we cannot estimate the models with unobserved heterogeneity using sampling weights, which is important given that adopters were over-sampled, we present the results assuming no unobserved heterogeneity. To assess the effect of ignoring unobserved heterogeneity, we re-estimate the models without sampling weights, and compare the estimated coefficients with and without random effects (see Tables A.1 in the Appendix A).

#### 4.2.1 Adoption

The probability of adoption increases steadily over time. Compared with season 2012B, adoption is three times as likely in 2013A, over four times as likely in 2013B and 2014A, six and a half times as likely in 2014B, over five times as likely in 2015A, and nearly eight times as likely in 2015B. These results are consistent with our descriptive statistics, which show adoption rapidly increasing over time. This time-path of adoption holds even after controlling for other factors.

Both formal delivery and informal dissemination significantly increase adoption. An additional direct marketing approach in the household’s sector increases the speed of adoption by 21%. An additional percentage point in the village level adoption rate, proxying for dissemination through social networks, increases the speed of adoption by about 3%. The ability to access and process information is positively correlated with adoption speed of iron-biofortified beans. An additional percentage point in the proportion of households that obtain information from extension in the village speeds adoption by about 1%. Households whose respondent has some primary or secondary education adopt about 45% faster than other households. Households in the top wealth tercile adopt 27% faster than the poorest households. Owning an additional piece of agricultural equipment increases the speed of adoption by 30%. Land area cultivated, however, is not correlated with adoption, suggesting that iron-biofortified bean is a scale-neutral technology. Finally, the speed of adoption varies significantly across varieties. All varieties are adopted more slowly than RWR2245. RWR2245 is likely the most popular at least partly because it has been the most heavily disseminated variety through the formal delivery approaches.

Table A.1 indicates that changes to the results of the adoption model are minimal when including random effects. Therefore, we conclude that any existing unobserved heterogeneity is not significant enough to alter our main findings.

#### 4.2.2 Disadoption

The likelihood of disadopting drops after the first season of growing an iron-biofortified variety. After two seasons of growing, the probability decreases by 57% compared to after one season (significant at 10%), and declines further in subsequent seasons. Thus, the longer households grow an iron-biofortified bean variety, the less likely they are to disadopt in each subsequent season.^7^

Payback is the only delivery approach significantly correlated with disadoption of iron-biofortified bean; adopters who live in a village where payback took place disadopt only 38% as quickly as households not located in such villages. While direct marketing, which reaches more households, increases the speed of initial adoption, targeting an area more intensively, which payback does, promotes more continuous adoption.

Female respondents disadopt only 65% as quickly as males. This could be due to the inclusion of women’s preferences in the development of the iron-biofortified bean varieties, and indicates that such efforts are working. While this difference could also be due to differences in market-orientation, our data show that men are not more likely to sell beans in general or iron-biofortified beans in particular, compared to women.

Knowledge in the form of education and experience in growing beans is also correlated with a lower speed of disadoption. Households whose respondent has some primary (secondary) education disadopt 66% (33%) as quickly as households whose respondent has no education. An additional year of experience cultivating beans reduces the speed of disadoption by 2%. This supports the hypothesis that more educated and experienced farmers may be more knowledgeable about bean management practices and better able to process and incorporate new knowledge about the variety and thus, more likely to obtain yields in line with expectations and less likely to disadopt.

Disadoption is similar across varieties apart from RWV1129 which is disadopted at a significantly slower rate than RWR2245. There is evidence that disadoption occurs both because varieties do not always meet household expectations and because planting material becomes unavailable. In total, 22% of disadopted iron-biofortified beans were disadopted because planting material was no longer available (Table A.2). This indicates that one in five disadopting households would have continued to grow iron-biofortified beans if planting material were more easily available. The remaining reasons for disadoption mostly pertain to dissatisfaction with variety traits, including yields (39%), other production characteristics (12%), consumption characteristics (2%), and market characteristics (22%). The reasons for disadopting RWV1129, the only variety disadopted more slowly than RWR2245, do not vary significantly from RWR2245.

Results with and without random effects are similar (Table A.1). The most notable change is a reduction in the statistical significance of the time dependence variables, indicating that not controlling for unobservable household heterogeneity may overestimate the effect of time on the disadoption decision. For the other significant covariates, the estimated coefficients are of similar size.

#### 4.2.3 Readoption

The probability of readopting drops dramatically after two seasons of discontinued use; a household is only 3% as likely to readopt after two seasons and 14% as likely after three seasons as it is after just one season of discontinued use. This result could reflect the seasonality of bean cultivation, where some households grow beans every other season (Asare-Marfo et al., 2016a). Households are equally likely to readopt after four or more seasons of discontinued use than they are after one season, likely due to grouping these seasons together.

Having an additional direct marketing approach in the sector more than doubles the speed of readoption, providing strong support that disadoption is partially driven by lack of available planting material. Informal dissemination is also positively correlated with readoption speed; a 1% increase in the previous-season village adoption rate increases the speed of readopting by 3%, although this is only significant at the 10% level.

Living an additional km away from an agrodealer or a city increases readoption speed by 3% (significant at 10%). This is contrary to expectations, but it may be that proximity to agrodealers or market centers makes it easier to switch varieties, reducing the likelihood households will readopt a variety they have stopped growing.

The varieties MAC44, MAC42, and RWV3317/RWV3006 are less likely to be readopted than RWR2245, given disadoption. Reasons for disadopting these varieties do not vary significantly from those cited for disadopting RWR2245.

Unobserved heterogeneity is present in our readoption model. Two differences in results between the models with and without unobserved heterogeneity are worth nothing (Table A.1). First, the impact of variety on readoption is smaller when household random effects are included. Second, we may also be underestimating the effect of agricultural equipment on readoption when not controlling for unobserved heterogeneity.

## 5 Conclusions and policy implications

The goals of this paper were to determine the most effective formal delivery approaches used so far in Rwanda to deliver iron-biofortified bean varieties and to assess the role of informal dissemination. Direct marketing within a sector speeds initial adoption and readoption while payback within villages (since replaced by seed swap) reduces disadoption. Policy makers should thus focus on these two approaches to improve long-term adoption of biofortified crops.

Our findings that social networks increase adoption indicate that, for biofortified crops, the positive effect of learning and obtaining planting material from neighbors outweighs potential negative effects of free-riding or strategic delay. This result is similar to that of McNiven and Gilligan (2012) who found that having other adopters of vitamin A biofortified orange sweet potato in farming households’ social networks improves their probability of adoption. This is encouraging, as informal dissemination will promote adoption, supplementing formal delivery at no cost. Policy makers should thus reach a broad area with biofortified crop dissemination rather than focus intensively on smaller areas, as informal networks will help to diffuse the crops when available.

Access to extension also plays a large role in initial adoption, indicating that either the general information provided by extension agents or their specific messaging about growing single-variety bean seeds is effective. This indicates that if policy makers continue to invest in the quality and coverage of extension services, adoption of biofortified crops will increase sustainably. Because women farmers play an important role in bean farming, are less likely to disadopt iron-biofortified bean varieties, and are less likely than men to cite agricultural extension officers as an information source (Asare-Marfo et al., 2016b), increasing women’s access to extension may be particularly helpful in promoting iron-biofortified bean adoption. In fact, our results indicate that the efforts undertaken so far to make iron-biofortified beans appeal to women have been effective, as women farmers are significantly less likely to disadopt the varieties than men. We also find that, while extension increases initial adoption, it plays no role in disadoption or readoption. Thus, once a household has its own experience with an iron-biofortified bean variety, additional knowledge about the varieties from official sources will likely not alter their adoption behavior.

Results of this paper can be used to inform delivery of biofortified crops in other countries. As biofortified crops continue to be released, policy makers can learn more lessons as to how to get these beneficial varieties to the people who need them most.

## 6 Declarations of interest

None.

## Figures and Tables

**Table A.1:** Complementary log-log results for adoption, disadoption and readoption models with and without unobserved heterogeneity/frailty.

	Adoption		Disadoption		Readoption	
	With unobserved heterogeneity	Without unobserved heterogeneity	With unobserved heterogeneity	Without unobserved heterogeneity	With unobserved heterogeneity	Without unobserved heterogeneity
	Hazard Ratio (Std. Err.)	Hazard Ratio (Std. Err.)	Hazard Ratio (Std. Err.)	Hazard Ratio (Std. Err.)	Hazard Ratio (Std. Err.)	Hazard Ratio (Std. Err.)
*Time dependence (base = 2012B/one season)*						
2013A/two seasons	2.914*** (1.204)	2.914*** (1.204)	0.547** (0.152)	0.439*** (0.084)	0.050** (0.062)	0.049** (0.051)
2013B/three seasons	5.136*** (1.981)	5.136*** (1.981)	0.472* (0.185)	0.353*** (0.103)	0.123* (0.153)	0.083** (0.087)
2014A/four seasons/four seasons or more	5.741*** (2.266)	5.741*** (2.266)	0.274** (0.162)	0.198*** (0.101)	2.882 (3.655)	0.800 (0.579)
2014B/five seasons or more	9.252*** (3.647)	9.252*** (3.646)	0.229* (0.185)	0.153*** (0.110)		
2015A	10.176*** (3.923)	10.176*** (3.923)				
2015B	20.721*** (8.258)	20.720*** (8.258)				
direct markets (# in sector)	1.131*** (0.037)	1.131*** (0.037)	1.064 (0.062)	1.054 (0.049)	2.736 (1.702)	1.588 (0.449)
payback (1 = in village)	1.194 (0.225)	1.194 (0.225)	0.638 (0.282)	0.677 (0.241)	0.628 (1.579)	0.905 (1.127)
seed swap (1 = in village)	1.293 (0.450)	1.293 (0.450)				
agrodealers (km)	0.995 (0.003)	0.995 (0.003)	1.002 (0.004)	1.001 (0.003)	1.031 (0.021)	1.017 (0.011)
multipliers (km)	1.001 (0.004)	1.001 (0.004)	0.996 (0.007)	0.996 (0.006)	0.963 (0.029)	0.984 (0.015)
village adoption rate	1.011** (0.004)	1.011** (0.004)	1.009 (0.007)	1.009 (0.006)	1.075* (0.040)	1.036** (0.015)
gender (1 = female)	1.153 (0.101)	1.153 (0.101)	0.632** (0.125)	0.688** (0.101)	0.158 (0.177)	0.452** (0.168)

*Education (base = no education)*						
some primary	1.311** (0.138)	1.311** (0.138)	0.829 (0.186)	0.859 (0.153)	1.781 (1.958)	1.063 (0.546)
some secondary or more	1.300 (0.222)	1.300 (0.222)	0.432** (0.165)	0.507** (0.148)	2.431 (4.616)	1.409 (1.224)
experience (years)	0.997 (0.003)	0.997 (0.003)	0.985** (0.007)	0.988** (0.006)	1.034 (0.034)	1.013 (0.015)
household size	1.053** (0.021)	1.053** (0.021)	1.006 (0.046)	1.017 (0.038)	1.085 (0.216)	1.063 (0.106)
share 0–5	0.683 (0.187)	0.683 (0.187)	2.222 (1.297)	1.780 (0.784)	1.266 (3.448)	0.696 (0.924)
share women	0.949 (0.266)	0.949 (0.266)	1.141 (0.681)	1.057 (0.518)	9.04 (27.852)	2.629 (4.066)

*wealth quint (base = 1)*						
2	1.089 (0.124)	1.089 (0.124)	0.811 (0.184)	0.844 (0.152)	1.567 (1.623)	1.278 (0.644)
3	1.397*** (0.160)	1.397*** (0.160)	0.842 (0.199)	0.871 (-0.164)	2.284 (2.443)	1.607 (0.749)
ag. equipment	1.118** (0.060)	1.118** (0.060)	1.119 (0.127)	1.125 (0.105)	4.262** (3.073)	1.864** (0.482)
cultivated land (100 m^2^)	1.000 (0.001)	1.000 (0.001)	0.997** (0.001)	0.996*** (0.001)	1.007 (0.006)	1.001 (0.003)
city distance (km)	1.007** (0.003)	1.007** (0.003)	1.005 (0.007)	1.005 (0.005)	1.056 (0.035)	1.022 (0.014)
ext. percent	1.006*** (0.002)	1.006*** (0.002)	0.996 (0.004)	0.996 (0.003)	0.978 (0.020)	0.996 (0.009)
social source			0.800 (0.150)	0.835 (0.128)	3.284 (3.302)	1.540 (0.647)

*Variety (base = RWR2245*^a^*)*						
MAC44	0.376*** (0.041)	0.376*** (0.041)	1.194 (0.267)	1.177 (0.227)	0.053** (0.079)	0.293** (0.151)
RWV3316	0.186*** (0.026)	0.186*** (0.026)	0.761 (0.249)	0.786 (0.222)	0.043 (0.088)	0.202 (0.175)
RWV3317	0.085*** (0.017)	0.085*** (0.017)	1.314 (0.472)	1.228 (0.357)		
RWV1129	0.099*** (0.018)	0.099*** (0.018)	0.912 (0.406)	0.876 (0.331)	0.514 (1.123)	0.866 (0.786)
RWR2154^a^	0.042*** (0.011)	0.042*** (0.011)	0.687 (0.373)	0.897 (0.384)	0.285 (0.681)	0.718 (0.873)
CAB2	0.069*** (0.015)	0.069*** (0.015)	0.828 (0.360)	0.921 (0.338)	0.329 (0.603)	0.398 (0.474)
RWV2887	0.063*** (0.014)	0.063*** (0.014)	2.079 (1.107)	1.706 (0.681)	0.363 (0.608)	0.714 (0.594)
MAC42	0.054*** (0.013)	0.054*** (0.013)	0.481 (0.267)	0.542 (0.265)	0.004 (0.022)	0.088 (0.196)
RWV3006	0.075*** (0.016)	0.075*** (0.016)	1.555 (0.628)	1.487 (0.484)	0.001** (0.002)	0.048*** (0.043)

Likelihood ratio test of rho = 0	${\bar{\chi }}^{2}$ = 0.00006 p = 0.490		${\bar{\chi }}^{2}$ = 1.880 p = 0.085		${\bar{\chi }}^{2}$ = 3.84 p = 0.025	

N	96,197	96,197	683	683	333	333

Note: Models were estimated without using sampling weights.

* = significance at 10%;

** = significance at 5%;

*** = significance at 1%. RWV3317 and RWV3006 had to be combined in the readoption model because RWV3317 perfectly predicted non-readoption.

a Bush variety

**Table A.2:** Reasons for disadoption by variety.

Variety	Low yield	Other production traits^a^	Consumption and storage traits^b^	Market traits^c^	Seed availability^d^	Other^e^	Don't Know	n
RWR2245	38.34%	12.10%	0.41%	7.98%	19.13%	20.50%	1.55%	129
MAC44	41.39%	11.35%	0.00%	7.83%	27.23%	11.18%	1.01%	62
RWV3316	51.18%	20.27%	1.73%	0.00%	19.04%	7.78%	0.00%	25
RWV3317	31.44%	1.23%	14.03%	3.60%	32.93%	16.77%	0.00%	20
RWV1129	39.74%	13.64%	0.00%	0.00%	15.04%	31.57%	0.00%	12
RWR2154	3.53%	42.12%	0.00%	24.74%	14.21%	15.40%	0.00%	8
CAB2	36.96%	16.02%	12.11%	7.87%	4.99%	22.04%	0.00%	15
RWV2887	50.43%	6.73%	0.00%	0.00%	40.46%	2.37%	0.00%	10
MAC42	36.17%	34.62%	0.00%	0.00%	16.16%	13.05%	0.00%	7
RWV3006	28.12%	0.00%	5.23%	0.00%	39.10%	27.55%	0.00%	15
All Varieties	38.47%	12.36%	1.94%	6.62%	22.20%	17.41%	0.99%	303

a Maturity period was too long/short; too many inputs required; poor drought resistance; poor flood resistance; poor pest resistance; poor disease resistance; labor intensive.

b Taste/quality was bad; the variety was difficult to prepare/cook; when prepared, variety tasted than expected; variety had a short/bad storage life.

c Seed was too expensive; did not fetch a good price at the market.

d Previous season’s harvest was all used; planting material no longer available in the nearby market.

e Crop management (rotation) practice; this variety is typically not grown in this season; I did not grow beans in this season.

## Appendix A

See Table A.1, Table A.2.

## Footnotes

1 Season A runs from September to February and Season B starts in March and ends in June (NISR, 2015).

2 Yields of the iron-biofortified varieties are similar to those of other improved varieties that were released during the same time period (Rwanda Agriculture Board, 2012). Improved bean varieties in Rwanda have been found to yield 80% on average higher than local varieties (Larochelle et al., 2015).

3 Disadoption (readoption) occurs sparingly after several seasons of use (discontinued use). We therefore group five seasons of use and more into one category for the disadoption model and four seasons of discontinued use and more into one category for the readoption model.

4 This could potentially bias the results if 2015B adopters (disadopters) made disadoption (readoption) decisions based on different criteria than previous adopters (disadopters), which we have no reason to believe is the case.

5 For the adoption model, AIC = 5,115,295; 5,123,593; and 5,121,468 for time dummies, log time and cubic time respectively. For the disadoption model, AIC = 610,568; 612,310; and 611,518 for dummies, log time and cubic time respectively. For the readoption model, AIC = 123,194; 136,127; and 122,967 for dummies, log time and cubic time, respectively.

6 ${\bar{\chi }}^{2}$ = 0.00006 & p-value = 0.490 for the adoption model; ${\bar{\chi }}^{2}$ = 1.88 & p-value = 0.085 for the disadoption model; ${\bar{\chi }}^{2}$ = 3.84 & p-value = 0.027 for the readoption model. This test had to be conducted without using sampling weights. Model results with and without sampling weights are similar, so we consider the test for unobserved heterogeneity to be valid.

7 Because some households grew more than one iron-biofortified bean variety, we estimated an alternative model specification in which a household that disadopted a variety but was still growing a different variety or immediately began growing a different variety was not considered a disadopter. Results for this specification were very similar to those of the disadoption model presented in Table 3.
