# Supplementary material for: Promoting rapid and sustained adoption of biofortified crops: What we learned from iron-biofortified bean delivery approaches in Rwanda
Source: Food Policy. 2019 Feb;83:271–84. doi: 10.1016/j.foodpol.2018.11.003 (PMC6472331; doi:10.1016/j.foodpol.2018.11.003)
Supplement: Supplementary data 1 [file mmc1.docx]

# Supplementary Material

S 1: Characteristics of iron-biofortified bean varieties. Source: Asare-Marfo et al., 2016a
